# Immunogenicity and efficacy of the novel cancer vaccine based on simian adenovirus and MVA vectors alone and in combination with PD-1 mAb in a mouse model of prostate cancer

**DOI:** 10.1007/s00262-016-1831-8

**Published:** 2016-04-06

**Authors:** Federica Cappuccini, Stephen Stribbling, Emily Pollock, Adrian V. S. Hill, Irina Redchenko

**Affiliations:** 1 The Jenner Institute, University of Oxford, Old Road Campus Research Building, Roosevelt Drive, Oxford, OX3 7DQ UK; 2 Present Address: Department of Surgery and Oncology, Imperial College London, Hammersmith Hospital, Du Cane Road, London, UK

**Keywords:** Prostate cancer, Vaccine, STEAP1, Immunogenicity, TRAMP model

## Abstract

Prostate cancer possesses several characteristics that make it a suitable candidate for immunotherapy; however, prostate cancer vaccines to date demonstrate modest efficacy and low immunogenicity. The goal of the present pre-clinical study was to explore the immunogenic properties and protective efficacy of a novel prostate cancer immunotherapy based on the heterologous prime–boost viral-vectored vaccination platform. The simian adenovirus, ChAdOx1, and modified vaccinia Ankara virus, MVA, encoding a prostate cancer-associated antigen, the six transmembrane epithelial antigen of the prostate 1 (STEAP1), induced strong sustained antigen-specific CD8+ T-cell responses in C57BL/6 and BALB/c male mice. Unexpectedly, the high vaccine immunogenicity translated into relatively low protective efficacy in the murine transplantable and spontaneous models of prostate cancer. A combination of the vaccine with PD-1 blocking antibody significantly improved survival of the animals, with 80 % of mice remaining tumour-free. These results indicate that the ChAdOx1–MVA vaccination regime targeting STEAP1 combined with PD-1 therapy might have high therapeutic potential in the clinic.

## Introduction

Prostate cancer (PCa) is the most common non-skin cancer in males and the second leading cause of cancer deaths, with an estimated 233,000 men diagnosed in 2014 and 29,480 deaths predicted in the USA [1]. Although there have been impressive advances made in recent years in the treatment of PCa, available therapies for advanced stages of the disease are still limited and their effectiveness is far from satisfactory. Therefore, the development of alternative therapies, aiming at activating host anti-tumour immunity using appropriate immunological targets, remains a priority.

Although promising, the use of therapeutic vaccination in cancer presents many challenges, with tolerance to self-antigens and active immunosuppressive mechanisms mounted by tumours being two major factors hampering cancer vaccine efficacy. The two most advanced PCa immunotherapies, the licensed product Sipuleucel-T [2] and ProstVac [3] currently being tested in Phase III trial, target two well-defined PCa antigens, prostatic acid phosphatase (PAP) and prostate-specific antigen (PSA), respectively.

In this study, we have evaluated an alternative PCa-associated antigen, the six transmembrane epithelial antigen of the prostate 1 (STEAP1), as a vaccine target in mouse models. STEAP1 is expressed only in the prostate among normal tissues and overexpressed in various cancer types including prostate, bladder, lung, ovarian cancer and Ewing sarcoma [4]. Its unique and restricted expression pattern and growing evidence of its role in PCa initiation and progression [5–7] make STEAP1 an excellent target for a PCa vaccine.

However, STEAP1 was shown to be poorly immunogenic when delivered as a DNA vaccine followed by Venezuelan equine encephalitis virus replicon particle (VEE VRP) boost [8]. In this study, we have deployed a highly immunogenic vaccination platform based on the two recombinant viral vectors, the simian adenovirus ChAdOx1 [9] and modified vaccinia virus Ankara (MVA), for the delivery of STEAP1 antigen and induction of STEAP1-specific cellular immune responses. This heterologous prime–boost regime has induced exceptionally strong T-cell responses in pre-clinical infectious disease models [10–12]. Also, ChAd–MVA vectors have been a very effective means of inducing CD8+ T-cells in humans—across 32 trials now in malaria, Flu, TB, HCV, HIV and Ebola using different inserts, and they have been safe in over 1200 vaccines, including adults, children and infants [13–17]. However, this approach has never been tested before in cancer settings.

In this study, for the first time a therapeutic vaccination strategy based on ChAdOx1–MVA prime–boost has been evaluated as a means of breaking tolerance and mediating tumour-protective efficacy in mouse transplantable and autochthonous models of PCa.

## Materials and methods

### Mice and cell lines

Six-week-old male C57BL/6 and BALB/c mice were purchased from Harlan, UK. TRAMP breeder female mice were purchased from Jackson Laboratories, and the colony was maintained in pure C57BL/6 background.

Mouse care and experimental procedures were carried out in accordance with the terms of the UK Animals (Scientific Procedures) Act Project License (PPL 30/2947) and approved by the University of Oxford Animal Care and Ethical Review Committee.

TRAMP-C1 cell line and HB-51 hybridoma (clone 28-8-6S) secreting mAb against MHC class I were purchased from the ATCC and maintained according to ATCC recommendations.

### ChAdOx1 and MVA viral vector construction

A DNA sequence encoding mouse STEAP1 antigen was obtained from Source BioScience, UK. The construction of ChAdOx1 vector was described previously [9]. The full-length mouse STEAP cDNA under a CMV immediate early promoter was sub-cloned from a pENTR plasmid into the E1 locus of the pBAC ChAdOx1-DEST genomic clone by Gateway™ cloning. The construct was then used to transfect HEK293A cells to generate the recombinant adenovirus expressing the antigen. The MVA-GFP shuttle vector drives the expression of STEAP1 under the P7.5 early/late promoter inserted at the thymidine kinase locus of MVA and the GFP from the fowlpox FP4b late promoter. The plasmid was transfected into MVA-infected primary chick embryo fibroblasts (CEFs), and recombinant virus was isolated by selection of GFP-positive plaques, amplified, purified over sucrose cushions and titrated in CEFs according to standard practice. The integrity, identity and purity of the viruses were confirmed by PCR analysis.

### In vivo studies

Immunogenicity of STEAP1 antigen expressed from the viral vectors was assessed in C57BL/6, BALB/c and TRAMP male mice. A dose of 10^7^ or 10^8^ infectious units (IU) of ChAdOx1 virus and 10^6^ or 10^7^ plaque-forming units (PFU) of MVA was given intramuscularly (i.m.) in a total volume of 50 µl per animal. The alternating immunisations with ChAdOx1.STEAP1 and MVA.STEAP1 vaccines were performed 1–3 weeks apart with a total number of 2–6 vaccinations. Transgene-specific immune responses in every animal were evaluated in blood 10–14 days after the prime and 7–10 days after the boost and in spleen at the point of sacrifice. Anti-mouse monoclonal antibodies against PD-1, PD-L1 and rat IgG2a were purchased from BioXCell and administered intraperitoneally at the dose of 300 µg per mouse weekly.

To assess vaccine tumour-protective efficacy in a transplantable tumour model, wild-type C57BL/6 male mice were inoculated with 2 × 10^6^ TRAMP-C1 cells in 100 µl PBS subcutaneously (s.c.) into the right flank. Upon establishment of palpable tumours, an immunisation course as described above was initiated. Tumour growth was monitored 3 times weekly, and animals were killed when tumour size reached 10 mm in any direction. Tumour volume was calculated as length (mm) × width^2^ (mm) × 0.5.

To assess vaccine tumour-protective efficacy in an autochthonous tumour model, an immunisation course of TRAMP male mice was initiated at 6–7 weeks of age. When mice reached 24–27 weeks of age, they were euthanised and the entire genitourinary tract (GUT) was dissected, weighed, paraformaldehyde (PFA)-fixed and paraffin-embedded.

### Measurement of STEAP1-specific immune responses by IFN-γ ELISPOT

An ex vivo IFN-γ ELISPOT assay was performed using Multiscreen-IP ELISPOT plates (Millipore) and mIFN-γ ELISPOT kit (ALP) (Mabtech). A mouse STEAP1 peptide library synthesised by Mimotopes (UK) consisting of 15-mer peptides overlapping by 10 amino acids and spanning the whole length of the STEAP1 protein was used for cell stimulation. Mouse PBMCs or splenocytes were incubated in triplicate wells with STEAP1 pools or individual peptides for 18–22 h or left unstimulated prior to detection of spot-forming cells (SFCs). ELISPOT plates were analysed using an AID ELISPOT counter (AID Diagnostika GmbH).

### Measurement of STEAP1-specific immune responses and MHC class I expression by flow cytometry

Mouse PBMCs or splenocytes were stimulated ex vivo with STEAP1 peptide pools in the presence of Golgi-Plug (BD) for 6 hours (h), labelled with CD4-FITC, CD8-APC, fixed-permeabilised in CytofixCytoperm buffer (BD) and stained with IFN-γ-PE antibody.

TRAMP-C1 and IFN-γ-treated TRAMP-C1 cells (100 U/ml for 48 h) were dissociated and incubated with HB-51 hybridoma supernatant or control medium followed by anti-mouse IgG-FITC secondary antibody. All antibodies used in this study were obtained from eBioscience. Flow cytometry was performed on a BD LSRII™ analyser and data analysed with FlowJo software.

### Analysis of STEAP1 expression on transcriptional and translational level

STEAP1 mRNA expression in TRAMP-C1 cells and thymi of C57BL/6 mice was detected by semi-quantitative reverse transcription PCR. Total RNA was extracted with RNeasy Plus minikit (Qiagen), and a total of 2 μg of RNA was used to synthesise the first single-strand cDNA using QuantiTect Reverse Transcription kit (Qiagen) according to the manufacturers’ guidelines. For RT-PCR amplification, the following priming pairs have been used: 5′-GGCATCCTCACCCTGAAGTA and 5′-AGCACTGTGTTGGCGTACAG for mouse β-actin, 5′-AGCACTGTGTTGGCGTACAG and 5′-TACAACCTGGAGGCCATCTC for mouse STEAP1, 5′-AGCACTGTGTTGGCGTACAG and 5′-AGCACTGTGTTGGCGTACAG for mouse 5T4. Transcripts were amplified using 98 °C denaturation, 60 °C annealing, and 72 °C extension for a total of 35 cycles.

STEAP protein expression in TRAMP-C1, LNCaP, PC3 and HEK293A cells infected with ChAdOx1 vectors (MOI = 10, 24 h) was detected by Western blotting. Cell lysates were obtained using RIPA buffer (Pierce Biotechnology) supplemented with protease inhibitor cocktail (Roche Diagnostics), and total protein concentration was determined by BCA protein assay (Thermo Fisher Scientific). For each cell line, 10 μg of total protein was subjected to SDS-PAGE and immunoblot analysis using rabbit anti-STEAP polyclonal antibody (Santa Cruz Biotechnology), followed by AP-conjugated secondary antibody (Jackson ImmunoResearch) and E SIGMAFAST BCIP/NBT detection system (Sigma).

### Immunohistochemistry (IHC) of TRAMP biopsies

Five-µm sections of PFA-fixed paraffin-embedded TRAMP GUTs were used in standard H&E staining or in IHC with anti-Ki-67 antibody (Cell Signalling) or anti-CD3 antibody (Abcam) and biotinylated secondary antibodies (Vector Lab). Immunoreactivity was visualised via avidin–biotin reagent and DAB substrate according to manufacturer’s instructions followed by counterstaining with Meyer’s haematoxylin.

Light microscopy pictures were obtained using a Leica DM5500B microscope and analysed with ImageJ software. Positivity for Ki-67 and CD3 markers was evaluated by calculating the ratio between brown-stained and blue-stained areas.

### Statistical analysis

Descriptive data were analysed with Prism 6.0 statistical software (GraphPad Software). All *P* values < 0.05 were considered statistically significant. Group comparisons were made by one-way ANOVA test or two-tailed Student’s *t* test. Survival curves were created with the Kaplan–Meier method, and the log-rank test was used to determine differences in survival between groups of mice. Each experiment presented in this manuscript is representative of at least 2 experiments using a minimum of 5 animals per group.

## Results

### ChAdOx1–MVA vaccination regime elicits strong STEAP1-specific CD8+ T-cells responses

**Fig. 1:**
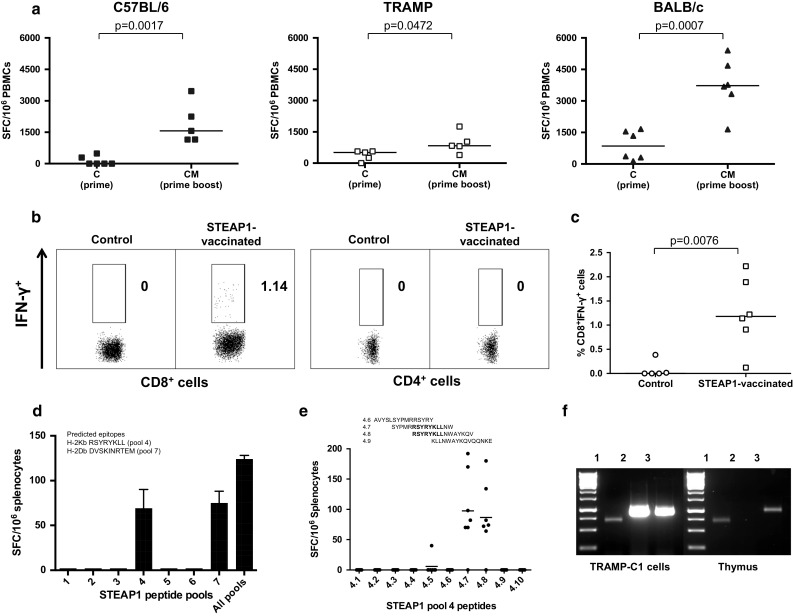
ChAdOx1–MVA prime–boost regimen induces strong STEAP1-specific CD8+ T-cell responses. Mice were immunised i.m. with 10^8^ IU of ChAdOx1.STEAP1 vector followed by 10^7^ PFU of MVA.STEAP1 3 weeks later. Representative data of 3 biological replicate experiments are shown. **a** Graphs show data of ex vivo blood ELISPOT performed after priming and boosting immunisations in C57BL/6 (*closed squares*), TRAMP (*open squares*) and BALB/c (*closed triangles*) male mice. *Bars* represent median responses as spot-forming cells (SFC) per 10^6^ PBMCs. *P* values are indicated. **b**, **c** Surface staining for CD4 and CD8 markers and IFN-γ intracellular cytokine staining (ICS) of PBMCs was performed in control and STEAP1-vaccinated TRAMP mice after MVA boost. **b** Graphs show the percentage of IFN-γ-positive CD8+ T-cells (*left panels*) and CD4+ T-cells (*right panels*) in one of the control and treated mice. **c** Relative proportion of STEAP1-specific CD8+ T-cell responses in peripheral blood of control and STEAP1-vaccinated mice after boost. Each symbol shows frequency of STEAP1-specific IFN-γ-secreting CD8+ T-cells in individual animals. Δ values in percentage are calculated subtracting values obtained in unstimulated cells in the two groups of mice. *Bars* represent median. *P* value is indicated. **d**, **e** Epitope mapping of STEAP1-specific responses. To dissect vaccination-induced T-cell responses, splenocytes isolated from ChAdOx1–MVA STEAP1-vaccinated mice were either stimulated with the total pool of STEAP1 peptides, all peptides separated into 7 pools (**d**), or individual overlapping peptides of pool 4 (**e**). The IFN-γ secretion was measured by ELISPOT assay. *Bars* represent SEM (**d**) and median (**e**). **f** Comparative expression of STEAP1 mRNA transcripts in TRAMP-C1 cells and murine thymus. TRAMP-C1 cell and thymic cDNA were amplified with primers specific for β-actin (*lane 1*), STEAP1 (*lane 2*) and 5T4 (*lane 3*)

In the first round of experiments, we set out to investigate whether immunological tolerance to STEAP1 can be broken by the ChAdOx1–MVA-based vaccine regime and to assess the magnitude of induced responses. C57BL/6, BALB/c and transgenic TRAMP male mice were primed with ChAdOx1.STEAP1 vaccine followed by MVA.STEAP1 boost 3 weeks later. An ex vivo IFN-γ ELISPOT assay using a pool of STEAP1 peptides covering the entire protein was performed on PBMCs after each vaccination. As shown in Fig. 1a, STEAP1-specific T-cell responses could be detected after a single priming immunisation in both mouse strains, and frequencies of antigen-specific T-cells significantly increased after MVA boost.

To assess the relative contribution of CD4+ and CD8+ T-cells in IFN-γ secretion, antigen-specific responses were analysed by flow cytometry. Representative results from one mouse post-MVA boost shown in Fig. 1b-c indicate that IFN-γ is predominantly secreted by CD8+ T-cells with approximately 1 % of lymphocytes in circulation being STEAP1-specific after boost vaccination.

In order to assess the breadth of the induced responses, splenocytes from vaccinated mice were exposed to the pool of STEAP1 peptides covering the entire protein as above, and to this pool dissected into 7 individual pools, each containing ten adjacent 15-mer peptides overlapping by 10 amino acids. As shown in Fig. 1d, only pools 4 and 7 were able to stimulate IFN-γ secretion. Putative CD8+ T-cell epitopes that could potentially bind to MHCI have been predicted by BIMAS software and validated previously [8]. A sequence alignment of the predicted MHCI epitopes with the 15-mer peptides constituting pools 4 and 7 demonstrated that these pools contain the predicted epitopes STEAP_186–193_ and STEAP_326–335_, respectively. Further dissection of pool 4 confirmed that vaccination-induced STEAP1-specific CD8+ T-cell responses were directed against the previously identified epitope STEAP_186–193_ RSYRYKLL (Fig. 1e).

Intrigued by the strength of the immune response elicited against a self-antigen, that was comparable with responses induced to pathogens by the same vaccination regime [13, 16], we have interrogated a murine thymus for STEAP1 expression. As shown in Fig. 1f, the STEAP1 mRNA transcript was not amplified from total thymic RNA, while a shared tumour antigen, 5T4, and β-actin were both amplified by RT-PCR. This finding suggests that precursors with STEAP1-specific TCR repertoire could have escaped negative thymic selection due to minimal thymic expression of STEAP1. Of note, a high intensity band corresponding to the STEAP1 mRNA transcript was detected by RT-PCR of total TRAMP-C1 cell RNA.

### ChAdOx1–MVA vaccination regime is protective in a transplantable tumour model

**Fig. 2:**
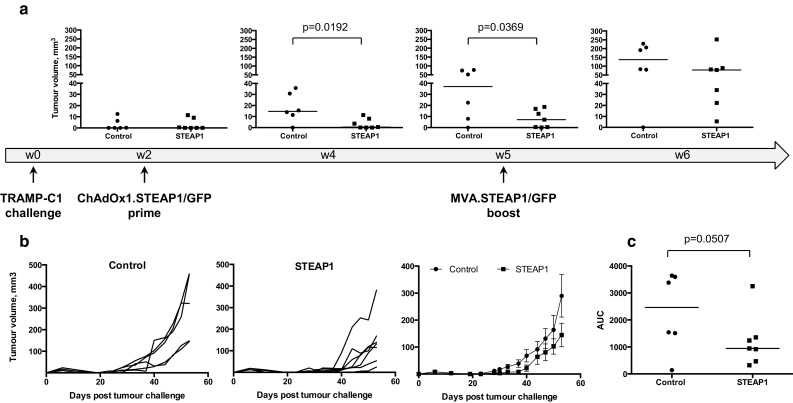
ChAdOx1–MVA prime–boost regimen reduces tumour growth rate in a transplantable tumour model. C57BL/6 mice were challenged subcutaneously with TRAMP-C1 cells and subsequently immunised i.m. at 3-week intervals with 10^8^ IU of ChAdOx1.STEAP1 and 10^7^ PFU of MVA.STEAP1, or equivalent doses of GFP-expressing vectors (control). Tumour size was measured 3 times per week, and volumes were calculated as described in “Materials and methods”. Representative data of 3 biological replicate experiments are shown. **a** Graphs show tumour volumes of individual mice in control and STEAP1-vaccinated groups expressed in mm^3^ calculated at weeks 2, 4, 5 and 6 post-TRAMP-C1 cell challenge. *Bars* represent median. **b** Tumour growth curves for each individual mouse in control (*left panel*) and STEAP1-vaccinated (*middle panel*) groups, and mean tumour volumes ± SEM comparison between the two groups (*right panel*) **c** Assessment of vaccine efficacy by end-point area under the curve (AUC) analysis (control group—*closed circles*; STEAP1 group—*closed squares*). *P* values are shown. *Bars* represent median

To determine whether strong STEAP1-specific T-cell responses could protect against tumour growth, mice were challenged s.c. with TRAMP-C1 cells. Upon establishment of palpable tumours, mice were primed with ChAdOx1 vectors expressing STEAP1 or control antigen GFP, and 3 weeks later boosted with the respective MVA vectors (Fig. 2a). The tumour volume was monitored at regular intervals throughout the experiment. As demonstrated in Fig. 2a, tumour growth was significantly delayed in treated mice compared to controls initially (weeks 4–5); however, over time the immune system started losing its ability to control tumour outgrowth (week 6). To quantitate and compare tumour growth kinetics, tumour growth curves of each mouse in control and treated groups were plotted (Fig. 2b). Single numerical values for individual curves were obtained for an area under the curve (AUC) analysis [18], which demonstrated a trend towards efficacy of STEAP1-targeting vaccine in delaying tumour progression (Fig. 2c).

### Optimisation of vaccination regime and PD-1 blockade improve protective efficacy of STEAP1 vaccine in a transplantable model

**Fig. 3:**
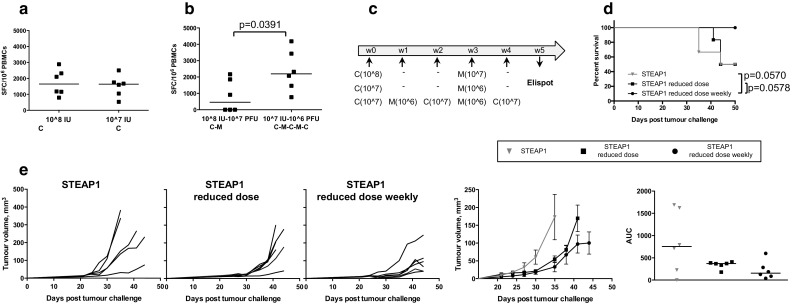
Optimisation of ChAdOx1–MVA vaccination regime. C57BL/6 mice were inoculated subcutaneously with TRAMP-C1 cells and randomised into three groups. Mice in group one were immunised i.m. with 10^8^ IU of ChAdOx1.STEAP1 and 10^7^ PFU of MVA.STEAP1 at 3-week intervals. Mice in group two received tenfold reduced doses of vectors, 10^7^ IU of ChAdOx1.STEAP1 and 10^6^ PFU of MVA.STEAP1 at 3-week intervals. Mice in group three were immunised with tenfold reduced doses at 1-week interval. Representative data of two biological replicate experiments are shown. ChAdOx1.STEAP1 is indicated as C, and MVA.STEAP1 is indicated as M. Blood samples were collected from mice in groups one and two 10 days post-prime **a** and in groups one and three 5 weeks post-prime–boost **b** for ex vivo blood ELISPOT to compare magnitude of STEAP1-specific responses. *P* value is shown. *Bars* represent median spot-forming cells (SFC) per 106 PBMCs. **c** Schematic representation of immunisation protocol. **d** Kaplan–Meier survival curves of the three groups of vaccinated mice. Log-rank (Mantel–Cox) test *P* values are shown. **e** Tumour growth curves for each individual mouse in the three groups, mean tumour volumes ± SEM comparison between the three groups, and assessment of vaccine efficacy by area under the curve (AUC) analysis at day 35 post-TRAMP-C1 cell challenge. *Bars* represent median

Our tumour challenge studies demonstrated that vaccine-induced immune responses can be tumour-protective at the onset of tumour development, but at a later stage the immune system fails to keep aggressive and fast-growing TRAMP-C1 tumours under control. We speculated that more frequent boosts with lower dose of vaccine may maintain better control of tumour growth. First, we tested the effect of vaccine dose reduction on the number of STEAP1-specific cells after prime with ChAdOx1.STEAP1. As shown in Fig. 3a, a tenfold reduction in the original dose resulted in similar frequency of antigen-specific T cells. Next, we compared the immunogenicity of three vaccination regimes: the original high dose of ChAdOx1 and MVA vectors given at 3-week intervals, the tenfold reduced dose of these vectors given at 3-week intervals and the reduced dose of these vectors given at 1-week interval (Fig. 3c). Interestingly, although the frequencies of T-cells after prime were comparable between 10^8^ and 10^7^ IU doses, 4 weekly boosts with reduced vaccine dose resulted in much stronger T-cell responses compared to the responses after 2 immunisations of high-dose vaccines given at 3-week intervals (Fig. 3b). The vaccination regime with reduced dose vectors administered at weekly intervals also translated into superior protection against tumour challenge compared to both standard and reduced doses given at 3-week intervals as demonstrated by the survival curves (Fig. 3d) and tumour growth kinetics (Fig. 3e).

**Fig. 4:**
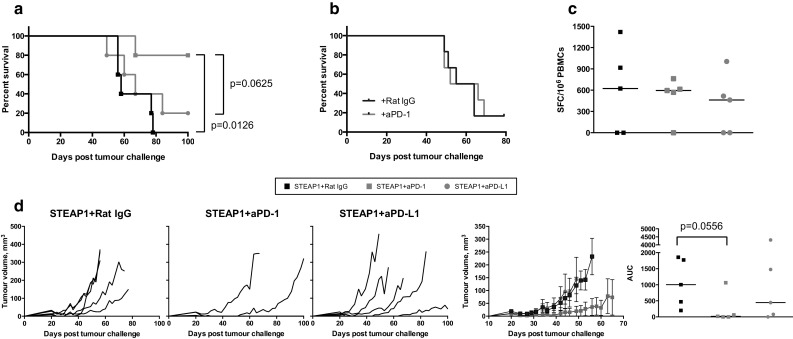
Anti PD-1 antibodies improve efficacy of STEAP1 heterologous vaccination in the transplantable tumour model. C57BL/6 male mice were inoculated s.c. with TRAMP-C1 cells and randomised into two groups. The first group was treated with the vaccine (10^7^ IU of ChAdOx1.STEAP1 and 10^6^ PFU of MVA.STEAP1 at 2-week intervals) combined with isotype control rat IgG antibody given i.p. weekly. The second and third groups received anti-PD-1 or anti-PD-L1 mAbs weekly in combination with the vaccine. In a different set of experiments, challenged mice were only given isotype control rat IgG antibody or anti-PD-1 mAb weekly. Tumour size was measured 3 times per week, and volumes were calculated as described in “Materials and methods”. Data of a representative experiment out of 2 biological replicates are shown. **a** Kaplan–Meier survival curves of the three groups of mice used in combination therapy experiments. Log-rank (Mantel–Cox) test *P* value is shown. **b** Kaplan–Meier survival curves of mice used in Ab only therapy. **c** Representative graph showing the effect of anti-PD-1 and anti-PD-L1 treatment on the magnitude of STEAP1-specific immune responses measured by ex vivo blood IFN-γ ELISPOT performed after ChAdOx1 prime–MVA boost in the three groups of mice. **d** Tumour growth curves for each individual mouse in the three groups, mean tumour volumes ± SEM comparison between the three groups and assessment of vaccine efficacy by area under the curve (AUC) analysis at day 49 post-TRAMP-C1 cell inoculation. *P* values are shown. *Bars* represent median

In an attempt to further increase vaccine efficacy, we also tested it in combination with PD-1/PD-L1 blocking antibodies. Checkpoint inhibitors have been remarkably successful in clinical trials in several cancer types, although so far there has been little evidence of efficacy in PCa patients, either as a monotherapy or in combination with a vaccine. A combinatorial approach has yet not been tested in a mouse PCa model, so we set out to fill in this gap. C57BL/6 mice were inoculated with TRAMP-C1 cells, and 1 week later a treatment course of the STEAP1 vaccine in combination with anti-PD-1 or PD-L1 mAbs was initiated. As a result, 80 % of mice receiving combination therapy with anti-PD-1 remained tumour-free, whereas all the other groups succumbed to tumours suggesting a synergistic effect of the vaccine and PD-1 blockade, resulting in tumour growth delay and significantly improved survival (Fig. 4a) as compared to STEAP1 vaccination combined with isotype control antibody or anti-PD-L1 mAbs. Importantly, anti-PD-1 treatment alone did not improve survival of tumour-challenged mice (Fig. 4b). Tumour growth kinetics of individual mice, mean tumour volumes per group and AUC analysis are shown in Fig. 4d. Of note, checkpoint blockade did not have any effect on the level of T-cells in the circulation as measured by ex vivo IFN-γ ELISPOT (Fig. 4c).

### ChAdOx1–MVA vaccination regime is modestly protective in an autochthonous model of PCa

**Fig. 5:**
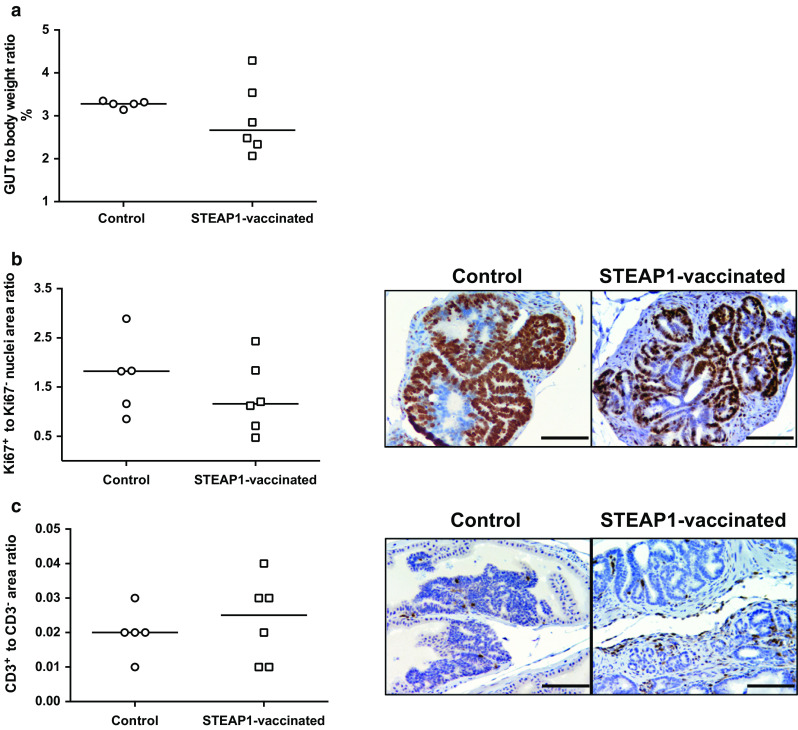
STEAP1 vaccination reduces tumour burden and increases T-cell infiltration in the prostate in spontaneous prostate cancer model. A 3-week interval vaccination was initiated on 6- to 8-week-old TRAMP mice using 10^8^ IU of ChAdOx1.STEAP1 and 10^7^ PFU of MVA.STEAP1. Immunised and control unvaccinated TRAMP mice were sacrificed at 25–27 weeks of age, and genitourinary tracts (GUTs) were dissected, weighed and embedded in paraffin. Representative data of 3 biological replicate experiments are shown. **a** Ratio of GUT wet weight to whole body weight in control and immunised TRAMP mice at 25–27 weeks of age. *Bars* represent median. **b**, **c** Immunohistochemistry staining of GUTs in STEAP1 vaccinated and control mice. Quantitative analysis and representative pictures of Ki-67 positive staining (**b**) and CD3 expression (**c**) of FFPE prostate tissue sections. *Bars* represent median. *Brown* regions indicate immunoreactivity; counterstain is haematoxylin (×200). *Scale bar* 100 μm

Next, we assessed the vaccine efficacy in the more physiologically relevant TRAMP mouse PCa model. TRAMP male mice uniformly develop prostate tumours due to expression of SV40Tag under the control of a prostate-specific androgen-regulated promoter [19]. By 22–25 weeks of age, the tumour progresses to advanced prostatic adenocarcinoma and mice die of metastatic disease 10–20 weeks later. Among vaccine efficacy readouts in the TRAMP model, the ratio of GUT to the whole body weight can be considered as a surrogate marker for tumour burden. To assess ChAdOx1–MVA vaccine, TRAMP male mice were immunised with STEAP1 vaccine at 3-week intervals starting at 6–8 weeks of age or left untreated until the age of 24–26 weeks. Mice were then sacrificed and GUTs were dissected and weighed. The whole body weight at the point of sacrifice was comparable in both groups (data not shown); however, GUT to body weight ratio in STEAP1-vaccinated mice was lower than in control animals (Fig. 5a). The trend towards less advanced cancer was also confirmed by histopathological analysis of prostate tissue sections (data not shown) and immunohistochemistry (Fig. 5b). As shown in Fig. 5b, the quantification of Ki-67 positive staining shows higher values in the control group as compared to STEAP1-vaccinated mice, which in turn demonstrated a slight increase in CD3+ tumour-infiltrating lymphocytes as compared to control mice (Fig. 5c).

### Inefficient antigen presentation by tumour cells, low-avidity T-cells and tumour adaptive immune resistance are underlying causes of modest vaccine efficacy

**Fig. 6:**
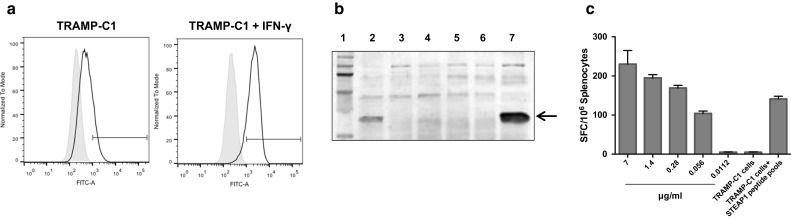
TRAMP-C1 cells are not able to efficiently present STEAP1 to T-cells. **a** Analysis of MHCI surface expression in TRAMP-C1 cells by flow cytometry. TRAMP-C1 cells or TRAMP-C1 cells pre-treated with IFN-γ were gently dissociated and stained with HB-51 hybridoma supernatant containing monoclonal anti MHCI antibodies (*black*), or with control medium (*grey*). Flow cytometry staining is shown as normalised histograms. **b** Western blot analysis of STEAP expression in LNCaP (*lane 2*), PC3 (*lane 3*), TRAMP-C1 (*lane 4*), HEK293A (*lane 5*), ChAdOx1.GFP infected HEK293A (*lane 6*), and ChAdOx1.STEAP1 infected HEK293A (*lane 7*) cells. *Lane 1* represents molecular weight markers. *Arrow* indicates STEAP1 bands. **c** Splenocytes were incubated with serial dilutions of STEAP1 pools, TRAMP-C1 cells or TRAMP-C1 cells preloaded with STEAP1 pools, and T-cell avidity measured by IFN-γ ELISPOT assay. *Bars* represent SD

Modest tumour-protective efficacy of the vaccine despite strong STEAP1-specific CD8+ T-cell responses has led us to investigate potential underlying mechanisms of this phenomenon. Firstly, the TRAMP-C1 cell line has been previously reported to express low levels of MHCI molecules [20] that could hamper presentation of STEAP1 epitopes on tumour cells. We confirmed this finding by incubating TRAMP-C1 cells with IFN-γ and staining them with anti-mouse MHCI mAb. Only 16.7 % of TRAMP-C1 cells naturally expressed MHCI molecules, while pre-treatment with IFN-γ raised MHCI expression to 87.6 % (Fig. 6a).

Although we have shown STEAP1 expression in TRAMP-C1 cells at a transcriptional level (Fig. 1d), we set out to investigate the expression of this antigen at the protein level. As shown in Fig. 6b, the band corresponding to STEAP1 protein is faint in TRAMP-C1 cells compared to LNCaP cells and ChAdOx1.STEAP-infected HEK293A cells used as positive control.

Poor presentation of STEAP1 antigen on the surface of TRAMP-C1 cells has also been confirmed in a functional assay. TRAMP-C1 cells used as APCs in an ex vivo ELISPOT assay have not induced IFN-γ secretion by splenocytes isolated from STEAP1-vaccinated mice. On the contrary, the same splenocytes could recognise TRAMP-C1 cells pulsed with STEAP1 peptides (Fig. 6c). In parallel, we tested the avidity of vaccination-induced effector cells by exposing them to serially diluted STEAP1 peptide pool. As shown in Fig. 6c, splenocytes require high concentrations of STEAP1 peptides to trigger IFN-γ secretion.

To conclude, in addition to low levels of MHCI–peptide complexes on the tumour cell surface, vaccination-induced T-cells are of low avidity, with both factors likely contributing to poor tumour control.

## Discussion

Various antigen delivery systems of poorly immunogenic prostate tissue-specific antigens, including the heterologous viral vector prime–boost approach, have been utilised in the past and elicited only modest immune responses [2, 3, 21, 22].

In the present study, we elected to evaluate a simian adenovirus prime–MVA boost vaccination platform, previously proved to be highly immunogenic in infectious disease settings, for its potential to break tolerance to self-antigens. This vaccination approach had been shown to induce unprecedentedly high CD8+ T-cell responses against pathogens [23–25] and also CD4+ T-cell responses. We have demonstrated for the first time the ability of the ChAdOx1–MVA vaccination strategy to induce strong sustained CD8+ T-cell responses to the tumour-specific self-antigen STEAP1 in murine models. In particular, ex vivo IFN-γ-secreting antigen-specific CD8+ T-cells could be detected after a single priming immunisation, with frequencies further increased by the MVA boost, with values comparable to the ones achieved by vaccinations against non-self-antigens, therefore confirming that the ChAdOx1–MVA vaccination strategy is an efficient means of inducing CD8+ T-cell responses against weakly immunogenic TAAs. For comparison, the highest frequency of STEAP1-specific T-cells following a heterologous vaccination regime based on DNA prime–VEE VRP boost was 60 SFC per million PBMCs [8]. Of note, STEAP1-specific CD4+ T-cells have been detected after ChAdOx1–MVA vaccination, but they were predominantly IL-2 secreting.

Surprisingly, there is general lack of studies investigating immunotherapies targeting STEAP1 antigen in PCa settings despite the fact that STEAP1 represents a promising antigenic target in this indication [5–7]. STEAP-specific immune reactivity has been demonstrated by two other heterologous vaccination strategies, DNA prime–MVA boost [26] and adenovirus prime–TRAMP cell lysate boost [27]; however, a direct comparison of the immunogenic potency of these two regimes with ChAdOx1–MVA is not possible as different assays were used to assess immunogenicity.

We have attempted to map CD8+ T-cell epitopes within the STEAP1 antigen. Notably, despite a large number of potential epitopes within STEAP1 predicted by MHCI binding software, the STEAP1-specific T-cell repertoire was narrow, with specificities directed only against two of the predicted epitopes. One of them, the H-2 Kb-restricted epitope RSYRYKLL (STEAP_186–193_), has also been identified by others [27].

Intrigued by unexpectedly strong immune responses induced against a self-antigen, we speculated that STEAP1 immunogenicity could stem from a breach in the central tolerance mechanism and escape from thymic selection, a phenomenon that has been observed for TRP-2 antigen [28]. To this end, we tested STEAP1 expression in murine thymus and found that STEAP1 expression is not detectable at mRNA levels. The same result has been reported by Hubert et al. [4] in relation to a human thymus. However, other reports describe the presence of very low levels of STEAP1 mRNA in the murine thymus [8, 29, 30]. This point remains controversial, but presumably if the thymic negative selection of T-cells with STEAP-reactive TCR does take place, it is not completely effective, as spontaneous STEAP-specific CD8+ T-cell responses have been detected in peripheral blood of PCa patients and in vitro re-stimulation has been described [31]. It appears that even if low levels of STEAP1 mRNA are present in the thymus, during the process of negative selection STEAP1-specific immature T-cells do not encounter their cognate antigen sufficiently to purge them from the T-cell repertoire and are allowed into secondary lymphoid organs and the periphery. The screening for thymic expression may be a promising approach for initial selection of potential target antigens for vaccine development.

In this article, we have shown that STEAP1 ChAdOx1–MVA vaccination platform delays growth of pre-established ectopic tumours at the early stages of tumour growth and modestly controls autochthonous tumours in TRAMP mice. Our data are in line with results published by others, where diverse prime/boost immunisation regimes targeting STEAP failed to significantly delay growth of established TRAMP tumours [8, 26, 27].

Overall, our results show that ectopic tumour growth can be delayed and disease progression can be inhibited in mice by STEAP1-specific T-cell responses, although tumour development could not be completely prevented despite the strong immunogenicity of the vaccine. There are a number of factors that could potentially contribute to the relatively inefficient control of neoplastic growth by vaccines. These include, but are not limited to, a natural immunosuppressive tumour microenvironment [32], active adaptive immune resistance [33, 34], impaired trafficking of immune cell into the tumour core [35] and impaired antigen processing and presentation by tumour cells [36]. We have investigated the potential impact of some of these factors on STEAP1 vaccine efficacy. TRAMP-C1 cells have been previously reported to express low levels of MHCI molecules [20, 37], and we demonstrate that upon IFN-γ treatment, MHCI surface expression dramatically increases in this cell line. Another important consideration is that natural expression of STEAP1 in TRAMP-C1 cells is low, and the number of the MHC–peptide complexes on the cell surface is not sufficient to activate STEAP1-specific responses in vitro. Similarly to other published data [27], here we show that TRAMP-C1 cells are able to efficiently stimulate IFN-γ secretion only when exogenously loaded with STEAP1 peptides. Thus, it is likely that TRAMP tumours are not recognised by vaccination-induced low-avidity T-cells because of their low MHCI and STEAP1 endogenous expressions. The low functional avidity of STEAP1-specific T-cells combined with the low level of MHCI–peptide complexes on target cells probably contributes to vaccine inefficiency. Indirect evidence supporting this hypothesis come from other PCa vaccine studies in TRAMP mice, where instead of relying on the natural expression of prostate-associated antigens in TRAMP tumours, investigators have chosen to use HA or SV40 T antigen as model tumour self-antigens [38, 39].

To improve vaccine efficacy, we explored the impact of reduced dose and shorter intervals of immunisations on tumour progression. We hypothesised that exposure to a lower amount of antigen could favour activation of high-avidity STEAP1-specific T-cells, which would be more efficient in targeting weakly immunogenic TRAMP-derived tumours. Generally, low-dose vaccination is known to stimulate low-frequency high-avidity T-cell responses, whereas high-dose peptide stimulates a larger frequency but low-avidity responses [40]. Importantly, our results show that a tenfold reduction in the vaccine dose has no impact on the magnitude of T-cell responses.

The rationale for decreasing intervals between ChAdOx and MVA vaccinations was based on several considerations. Firstly, long intervals between immunisations proved to be the most efficacious in mouse malaria challenge studies [41] might not be optimal in murine cancer models. Assuming that, following immunisations against a weakly immunogenic self-antigen, the pool of activated T-cells is smaller and cell activation status is lower, shorter intervals between immunisations may be beneficial. Secondly, the rapid tolerisation of activated T-cells in circulation when they reach tumour sites [38, 42] and the aggressiveness of the TRAMP tumour model suggest that frequent fresh supply of effector cells could result in a better tumour growth control. In fact, several publications demonstrated that good tumour-protective efficacy was achieved with one-week interval immunisations [39, 43]. To conclude, our experimental data confirmed that a reduced vaccine dose and shorter intervals between boosts elicit strong and sustained IFN-γ responses and increase vaccine efficacy.

Currently, the tumour adaptive immune resistance and checkpoint blockade are under intense investigation [33, 34, 44], and anti-tumour activity of vaccines was found increased when combined with antibodies blocking PD-1 or PD-L1 [45–47].

The clinical activity of checkpoint monotherapy has been confirmed in melanoma, renal and lung cancer. This approach may be also advantageous for targeting PCa. In this study, we have tested whether it is possible to optimise our strongly immunogenic but moderately efficacious vaccination platform intervening on the PD-1/PD-L1 axis. Our results show that blockade of PD-1 enhances the efficacy of STEAP1 vaccination in the subcutaneous tumour model significantly improving survival of STEAP1-vaccinated mice compared to STEAP1 vaccine or anti-PD-1 therapy alone. Interestingly, in a recent study Rekoske et al. [47] found that immunisation of tumour-bearing mice with a modified, more immunogenic DNA vaccine elicited a surprisingly limited anti-tumour effect relative to the native vaccine. They demonstrated that antigen-specific CD8+ T-cells from mice immunised with the optimised construct expressed higher PD-1 and, partly in line with our observations, anti-tumour activity of the optimised vaccine increased when combined with PD-1 or PD-L1 blocking antibodies. Of note, we could not detect any effect of anti-PD-1 mAb treatment on the magnitude of STEAP1-specific T-cell responses in circulation, suggesting that the observed synergistic effect could be taking place at the tumour site [48, 49]. We should also mention that in our hands, no beneficial effect was obtained using anti-PD-L1 antibody therapy in a combinatorial approach, possibly reflecting the fact that PD-L1 is not upregulated in this tumour model, similarly to human PCa [50].

To conclude, data presented in this study demonstrate that ChAdOx1–MVA-based vaccination strategy targeting prostate-associated antigen STEAP1 is able to elicit strong sustained STEAP1-specific immunity in mice and confers partial tumour protection in transplantable and spontaneous PCa mouse models. Also, our vaccination regime in combination with PD-1 therapy significantly improves survival in tumour-bearing animals. We believe that STEAP1 ChAdOx1–MVA vaccination has high therapeutic potential and an improved efficacy in pre-clinical animal models could rapidly translate into clinical use.

## Abbreviations

AUC: Area under curve
CEF: Chick embryo fibroblast
GUT: Genitourinary tract
IU: Infectious unit
MVA: Modified vaccinia Ankara
PAP: Prostatic acid phosphatase
PCa: Prostate cancer
PD-1: Programmed cell death-1
PD-L1: Programmed cell death ligand-1
PFA: Paraformaldehyde
PSA: Prostate-specific antigen
SFC: Spot-forming cell
STEAP1: Six transmembrane epithelial antigen of the prostate 1
TRAMP: Transgenic adenocarcinoma of the mouse prostate
VEE VRP: Venezuelan equine encephalitis virus replicon particle
